# Rapid Determination of Low Heavy Metal Concentrations in Grassland Soils around Mining Using Vis–NIR Spectroscopy: A Case Study of Inner Mongolia, China

**DOI:** 10.3390/s21093220

**Published:** 2021-05-06

**Authors:** Aru Han, Xiaoling Lu, Song Qing, Yongbin Bao, Yuhai Bao, Qing Ma, Xingpeng Liu, Jiquan Zhang

**Affiliations:** 1 School of Environment, Northeast Normal University, Changchun 130024, China; arh690@nenu.edu.cn (A.H.); baoyb924@nenu.edu.cn (Y.B.); maqing95708@163.com (Q.M.); liuxp912@nenu.edu.cn (X.L.); 2 Laboratory for Vegetation Ecology, Ministry of Education, Changchun 130024, China; 3 State Environmental Protection Key Laboratory of Wetland Ecology and Vegetation Restoration, Changchun 130024, China; 4 College of Tourism and Geographical Science, Baicheng Normal University, Baicheng 137000, China; lxl7302@163.com; 5 College of Geographical Science, Inner Mongolia Normal University, Hohhot 010022, China; qingsong@imnu.edu.cn (S.Q.); baoyuhai@imnu.edu.cn (Y.B.)

**Keywords:** soil spectral information, spectral transformation, heavy metal, Vis–NIR, organic matter

## Abstract

Proximal sensing offers a novel means for determination of the heavy metal concentration in soil, facilitating low cost and rapid analysis over large areas. In this respect, spectral data and model variables play an important role. Thus far, no attempts have been made to estimate soil heavy metal content using continuum-removal (CR), different preprocessing and statistical methods, and different modeling variables. Considering the adsorption and retention of heavy metals in spectrally active constituents in soil, this study proposes a method for determining low heavy metal concentrations in soil using spectral bands associated with soil organic matter (SOM) and visible–near-infrared (Vis–NIR). To rapidly determine the concentration of heavy metals using hyperspectral data, partial least squares regression (PLSR), principal component regression (PCR), and support vector machine regression (SVMR) statistical methods and 16 preprocessing combinations were developed and explored to determine an optimal combination. The results showed that the multiplicative scatter correction and standard normal variate preprocessing methods evaluated with the second derivative spectral transformation method could accurately determine soil Cr and Ni concentrations. The root-mean-square error (RMSE) values of Vis–NIR model combinations with PLSR, PCR, and SVMR were 0.34, 3.42, and 2.15 for Cr, and 0.07, 1.78, and 1.14 for Ni, respectively. Soil Cr and Ni showed strong spectral responses to the Vis–NIR spectral band. The R^2^ value of the Vis–NIR-based PLSR model was higher than 0.99, and the RMSE value was 0.07–0.34, suggesting higher stability and accuracy. The results were more accurate for Ni than Cr, and PLSR showed the best performance, followed by SVMR and PCR. This perspective has critical implications for guiding quantitative biogeochemical analysis using proximal sensing data.

## 1. Introduction

Although coal mining promotes local economies, it also causes serious environmental pollution [1,2,3]. Heavy metals in coal and coal spoil can enter soil through various routes, leading to the contamination of soil around mining areas [4,5]. Soil heavy metal contamination not only increases food safety risks, but also directly threatens human health [6]. In particular, heavy metals in the human body can undergo a latent accumulation process, and when their content exceeds the maximum capacity of the human body, various diseases may arise. Heavy metal poisoning increases the likelihood of liver, kidney, stomach, and nerve tissue damage, leading to teratogenesis, carcinogenesis, and mutagenesis, in serious cases. Therefore, with increasing focus on environmental issues and ecological conservation, the real-time monitoring of soil around mining areas has become an urgent requirement.

A critical aspect of the effective prevention and control of soil heavy metal pollution is rapidly acquiring accurate information on the concentration and spatial distribution of heavy metals. However, traditional methods of monitoring and identifying soil heavy metals involve field collection and lab analysis of samples [7]. Although such methods provide highly accurate results, they are laborious, costly, and time-consuming in large-scale monitoring of soil heavy metal concentrations. Therefore, it is difficult to describe dynamic changes of pollution elements on a large scale using traditional methods because they have spatial and temporal limitations. With the advantages of rapidity, non-destructivity, and high spectral resolution, hyperspectral proximal sensing has momentous functions in quantitative soil monitoring [8,9,10]. Considering its research value and practical significance, hyperspectral proximal sensing was introduced into the rapid determination of soil heavy metal concentration around mining areas. Vis–NIR has been used to determine heavy metal concentrations in soils since 1997 [11]. The Vis–NIR reflectance of soil can provide information on the accumulation properties of heterogeneous combinations of organic matter (OM), soil moisture, particle size and distribution, iron oxide, soil mineralogy, and parent material.

The accuracy of models based on hyperspectral data for determining soil heavy metals is affected by different physicochemical properties of different types of soil, differences in heavy metal content, different methods of data preprocessing, spectral resolutions, band ranges used, and different forms of transformations. In most instances, preprocessing variables can effectively eliminate and reduce multicollinearity and randomness between spectral bands to improve the accuracy and stability of the model [12]. Current approaches toward improving modeling accuracy can be mainly classified as follows: (1) Using a band combination approach based on comprehensive information associated with spectral signals, and transforming multiband reflectance by certain mathematical processes, to highlight major information and minimize minor information. This approach could be applied to eliminate the effect of multicollinearity among variables, reduce effective signal-to-noise ratio (SNR), and eliminate background interference, thus enhancing useful information and suppressing interference [13,14]; (2) The response of spectral bands varies widely among soil properties. Many researchers have removed noise generated during spectral analyses using the spectral information of pretreated raw soil and removed the effects of baseline and overlap to a certain extent, with good performance of the constructed models [15,16]. All preprocessing techniques aim to reduce un-modeled variability in data, which is necessary for enhancing spectral information [17,18].

Another important factor affecting the predictive capacity of models is band selection [19]. Soil reflectance is only loosely associated with the concentration of transition elements [20]. At low concentrations, heavy metals in soil cannot be identified directly with Vis–NIR reflectance [21,22]. Studies have demonstrated that Fe oxides, clays, and OM exhibit spectral activity in Vis–NIR spectra [23,24]. Therefore, soil spectral reflectance can reflect the concentration of heavy metals in soil according to the correlation between contaminant elements and active spectral components in soil [8,22,25]. Heavy metals and soil components, such as soil organic matter (SOM), clay minerals, and Ferromanganese (Fe-Mn) oxide, exhibit prominent adsorption characteristics, enabling the indirect prediction of heavy metal concentration from soil reflectance [26,27]. The adsorption and retention of heavy metals by spectrally active components in soil vary with the contamination elements and soil conditions. Some scholars used the adsorption relationship of SOM, clay minerals, and heavy metals in soil to indirectly establish an inversion model for heavy metals in soil [28,29,30,31]. Via simultaneous adsorption–desorption analyses of Cd, Cr, Cu, Ni, Pb, and Zn, researchers found that OM has stronger adsorption for Ni, and clays containing kaolinite have strong retention for Ni [32]. Moreover, studies investigating the behaviors of Ni and Zn in adsorption and desorption experiments have found that Ni binds to clay and SOM with relatively high intensity [33,34]. Although heavy metals with low concentrations have no spectral characteristics in the Vis–NIR region, the concentrations of non-characteristic elements in soil can be predicted by their correlations with OM, clay minerals, and iron oxides [22,35,36]. The determination of heavy metal concentration using hyperspectral proximal sensing is affected not only by the spectral band, but also by the original spectral noise. As a consequence, it is necessary to select specific treatment methods and modeling variables according to the spectral characteristics of the soil.

The application of spectroscopy is to establish the mathematical relationship between spectral and soil properties based on a calibration model. Once a calibration model is developed, it can be used to predict the chemical or physical properties of unknown samples. For this purpose, different multivariate statistical methods can be used. The most commonly used methods include multiple linear regression (MLR) [37], principal component regression (PCR) [38], partial least squares regression (PLSR) [39], artificial neural networks (ANNs) [40], support vector machine regression (SVMR) [41], and regression trees [42]. There is no best method because each one has its advantages and drawbacks. For example, PCR and PLSR have the advantage of handling data multicollinearity compared to MLR, but they are only capable of estimating the linear relationship between spectral and soil properties. On the contrary, the latest techniques, ANN and SVMR, can manage the nonlinear behavior of soil reflectance [23]. In particular, SVMR is based on the statistical learning theory [43] and exhibits high performance in training calibration models with few samples. However, there is no specific conclusion regarding the most effective and accurate method.

This study aimed to rapidly determine the concentration of heavy metals using spectral bands associated with SOM and Vis–NIR in soil, taking different grassland soils around two coal mining areas as the research objects. PLSR, PCR, and SVMR statistical methods and 16 preprocessing combinations were developed and explored to determine the optimal combination. The objective was to evaluate the predictability of Cr and Ni concentrations using a Vis–NIR spectroscopy technique, by considering the entire reflectance spectrum (350–2500 nm) and only that related to SOM absorption (600–800 nm). To achieve this, the statistical modeling methods of PLSR, PCR, and SVMR, and 16 preprocessing combinations were tested to determine an optimal combination that provides accurate estimation models. The findings of this study will provide a reference for future related research.

## 2. Materials and Methods

A method using Vis–NIR and spectral bands associated with OM is proposed for the determination of low heavy metal concentration in soil. The influence of different preprocessing and statistical methods on the accuracy of the determination model was investigated to achieve the most suitable effect. In order to explore the most suitable model combination for determination, 201 absorption spectral bands associated with SOM and 2150 Vis–NIR spectral bands were extracted as independent variables to establish the estimation model, considering PLSR, PCR, and SVMR for soil Cr and Ni concentrations. The coefficient of determination (R^2^) and RMSE represent the stability and accuracy of the estimation model, respectively. Three-quarters of the measured soil reflectance spectra were grouped into a calibration set, and the remaining one-quarter of soil reflectance spectra were used as validation samples; the calibration and validation sets comprised 27 and 10 samples, respectively. Data from the other 9 sampling points in the study area were used to validate the PLSR estimation model for Cr and Ni concentrations.

### 2.1. Study Area

**Figure 1:**
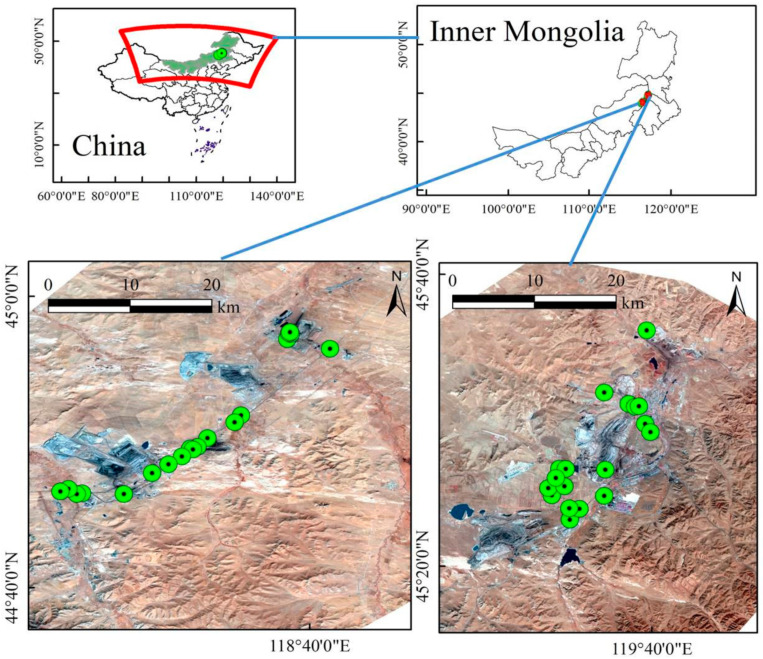
Study areas and sampling sites.

In this study, the Huolinhe open cast coal mine and Baiyinhua coal mine were selected as the research objects. Figure 1 presents a schematic diagram of the study area. The base map was the Landsat8 OLI image of the study area, which was downloaded from the geospatial data cloud [44]. Study area 1 is the Huolinhe coalfield, which is located in Tongliao City, Inner Mongolia Autonomous Region. It is the largest open cast coal mine with the highest production among modern coal mines in China; it has a reserve of 13.28 Gt. The Huolinhe coalfield was the first modern open cast coal mine in Asia, with an annual production capacity of 10 Mt. The coalfield is 9 km wide and 60 km long, with a total area of 540 km^2^. There are 9 minable coal seams, with a total thickness of 81.7 m. It stores 13.1 Gt of high-quality lignite, which is 9-fold greater than that of the Fushun Coal Mine, and 4-fold greater than that of the Datong coal mine, and has achieved an annual production capacity of 15 Mt. The geographical coordinates are 119°10’–119°38’ E and 45°11’–45°34’ N. Study area 2 is Baiyinhua coal mine, located in West Ujimqin Banner, Inner Mongolia Autonomous Region, China. Baiyinhua has 4 open cast mines and is one of the top ten coalfields in the Inner Mongolia Autonomous Region, with proven reserves of 14.07 Gt. There are 3 coal groups in the coal seam, with an average thickness of approximately 16 m, which are high-quality, medium-ash, low-sulfur lignite.

### 2.2. Sample Collection and Processing

In October 2018, soil samples were collected from grasslands around the two coal mining areas. The plum blossom point distribution method was used to arrange points around the mining area [45]. Soil samples were collected from 0 to 10 cm of the soil layer at five points in each sampling site. The location of each sampling site was recorded using a handheld Global Positioning System (GPS). Approximately 1 kg of each soil sample was collected in a clean plastic bag, sealed, and numbered; a total of 37 soil samples were collected. The samples were dried, pulverized, and sieved (100 mesh sieve). Each sample was divided into two parts, one for chemical analysis of SOM, heavy metals, and water content, and another for spectral analysis in the laboratory.

Soil pH was measured using a pH meter in 1:2.5 (mass to volume ratio) soil and deionized water suspensions. SOM was determined using potassium dichromate. For sample preparation, microwave acid digestion apparatus was used, and the samples were digested with HNO_3_-HF-HClO_4_ before analysis. The metal concentration in the samples was determined through inductively coupled plasma atomic emission spectrometry (ICP-AES, Optima 2000DV) [46,47,48].

### 2.3. Acquisition of Indoor Spectral Data of Soil Samples

In this study, an ASD FieldSpec4 spectroradiometer was used for spectral data acquisition. The wavelength range was 350–2500 nm, the spectral resolutions were 3 nm at 700 nm, 30 nm at 1400 nm, and 30 nm at 2100 nm, and the sampling intervals were 1.4 nm at 350–1000 nm and 2 nm at 1000–2500 nm. Soil samples were directly measured by a hand-held soil probe with an embedded light source. The light source was a 50 W halogen lamp. The spectrometer was calibrated by the standard white BaSO_4_ panel before determination. The sample was placed in a 6 cm diameter and 1.5 cm deep dish, and spectral reflectance was measured after scraping the soil surface. During measurements, the sample dish was rotated 90° for three turns. From each soil sample, ten spectral curves were collected in replicates. The mean value was taken as the final reflectance, and a standard white BaSO_4_ panel calibration was performed every 15 min. The spectrometer resampled the spectral data at 1 nm intervals during the output values [49].

### 2.4. Data Processing

#### 2.4.1. Continuum-Removal Method

The following process was applied to the resampled data. The CR method is a spectroscopic analysis approach for removing unrelated background features and enhancing absorption characteristics of interest [50]. The CR method can normalize the spectral reflectance to 0–1 while maintaining the same background, effectively highlighting absorption valleys and reflection peaks of the spectral curve. Therefore, the resampled data were first CR processed.

#### 2.4.2. Spectral Data Preprocessing and Transformation

The reflectance (R) and CR were preprocessed by smoothing with the Savitzky–Golay filter (fitting times: 2, window width: 9) [51]. Spectral preprocessing can be applied to remove the effects of scattering between soil samples. Spectral transformation methods can eliminate noise generated by spectral data, highlight spectral valleys and peaks, and enhance the response of heavy metal elements in soil spectra. The R and CR after SG smoothing were used for preprocessing using the normalization (NOR), multiplicative scatter correction (MSC) [52], and standard normal variate (SNV) [53] methods. Finally, the processed data were subjected to First Derivative (FD), Second Derivative (SD), and Reciprocal Logarithm (log(1/R)) spectral transformations. In this manner, 16 methods of preprocessing were evaluated, as shown in Table 1.

### 2.5. Extraction of Absorption Spectral Band Associated with Organic Matter 

**Figure 2:**
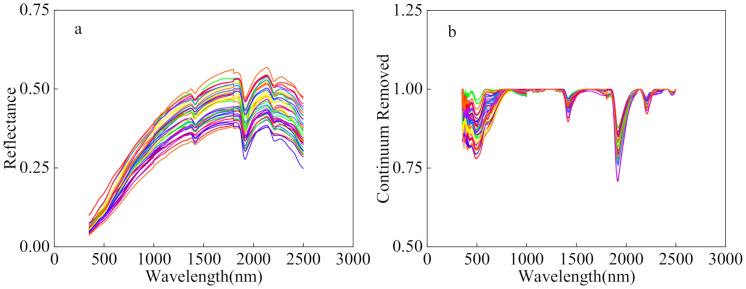
Laboratory spectral data: (**a**) raw spectral; (**b**) continuum-removal (the colored lines represent different sampling points).

Previous studies have shown that the main components of soils, such as SOM and clay minerals, have distinct absorption characteristics, and much work has been conducted on their quantitative determination [54,55,56]. The impact of SOM was mainly reflected in the Vis–NIR wavelengths, with the greatest impact in the 600–800 nm band [57]. The raw spectral curves of soil in Figure 2a showed the occurrence of prominent absorption valleys at 1400 and 1900 nm, i.e., water absorption bands, which are usually considered to be related to soil water content. The absorption band was extracted based on the CR spectra, and the absorption band was more pronounced after CR (Figure 2b). The maximum absorption band and absorption width were determined according to the absorption depth, and the SOM characteristic band was extracted at a half-width interval in the absorption region to ensure that the selected spectral band had a strong absorption capacity. Therefore, the absorption spectra at 600–800 nm were considered to be associated with SOM.

### 2.6. Modeling

#### 2.6.1. Partial Least Squares Regression (PLSR)

In this study, PLSR was used for predicting heavy metal concentrations in soil. PLSR is widely applied in many fields and can be regarded as a reference method. It is a new multivariate statistical regression method that integrates canonical correlation analysis, principal component analysis, and multiple linear regression analysis. The method can use all effective data to construct a model and extract the maximum information reflecting data variation; moreover, it has a good prediction function [58] and a unique advantage in handling variables with high internal correlation. Therefore, PLSR has been receiving increasingly more attention in the field of hyperspectral proximal sensing. This method has been well established in the construction of predictive models for spectral and crop physicochemical parameters and soil information.

#### 2.6.2. Principal Component Regression (PCR)

PCR is an unsupervised pattern recognition algorithm. When establishing a multiple linear regression equation, multicollinearity exists among variables, due to which the coefficients of some independent variables become extremely unstable. When increasing or decreasing variables, the coefficients of independent variables may change significantly, and even lead to symbols inconsistent with the actual situation, leading to inconsistencies in the established regression equation. The PCR algorithm attempts to reduce the dimension of independent variables in order to solve the multicollinearity problem among independent variables, which can enhance relevant information about components and filter out some noise signals that cause interference [59]. This algorithm can extract the principal component containing basic information of the sample and use linear transformation to transform the original high-dimensional data into a tablespace. The new principal component band images obtained by the transformation are not related to each other, and there are significant differences between the data. With increasing eigenvalues, the proportion of the new variables obtained by the transformation to express the original data also increases.

#### 2.6.3. Support Vector Machine Regression (SVMR)

SVMR is a class of generalized linear classifiers for binary classification, which is an important application of support vector machines (SVMs). SVMR has only one class of sample points in the end, and it seeks an optimal hyperplane without maximizing the distance between two or more classes of sample points to the nearest sample point in the hyperplane, as in SVM. On the contrary, SVMR attempts to minimize the distance to the farthest sample point in the hyperplane [60]. It is a new modeling method that improves the generalization ability through the principle of structural risk minimization and better solves various practical problems, such as small samples, nonlinearity, high dimensionality, and local minima. It is emerging as a powerful tool for solving traditional problems such as “dimensional disaster” and “overlearning” [61]. 

Unscrambler X 10.4 (Unscrambler version X 10.4, CAMO, Trondheim, Norway) and Origin 2021 (for mapping and processing) were used for elemental concentration analysis and monitoring of soil heavy metal contamination.

## 3. Results

### 3.1. Description of Soil Samples

The soil was alkalized meadow soil with a pH of approximately 8–8.5. Descriptive statistical analyses of the calibration/validation set (Table 2), including the calculations of mean, standard deviation (std), kurtosis, skewness, coefficient of variation (CV), maximum values, and minimum values, were performed to analyze the soil in the study area. The average values of Cr, Ni, SOM, and water content were 16.59, 5.78, 2.93, and 5.06, respectively. The concentrations of heavy metals were higher than background values in only a few instances, and all mean concentration values were below the national secondary standard values [62]. The concentration ranges of Cr and Ni were 8.02–24.12 mg·kg^−1^ and 0.01–10.22 mg·kg^−1^, respectively. The maximum values of Cr and Ni were 1.14- and 1.01-fold greater than their background values, respectively, indicating a certain enrichment of heavy metals in surface soil. The K–S test indicated that soil data followed a normal distribution. The skewness of Cr and Ni were negative at −0.24 and −0.56, respectively, indicating that high-frequency ranges occurred in areas of high concentrations. The kurtosis of Cr and Ni were positive at 0.11 and 0.70, respectively, indicating that they were more concentrated than the normal distribution.

### 3.2. Model Construction and Evaluation

#### 3.2.1. Estimation Model Based on R and CR Spectral Data

**Figure 3:**
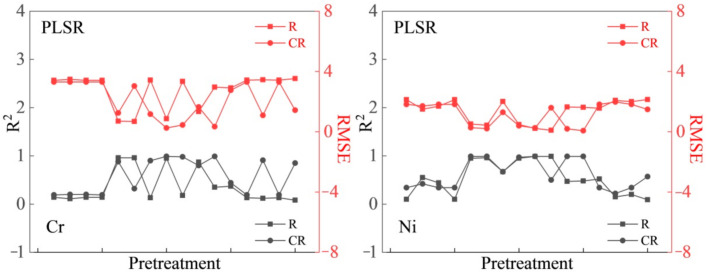

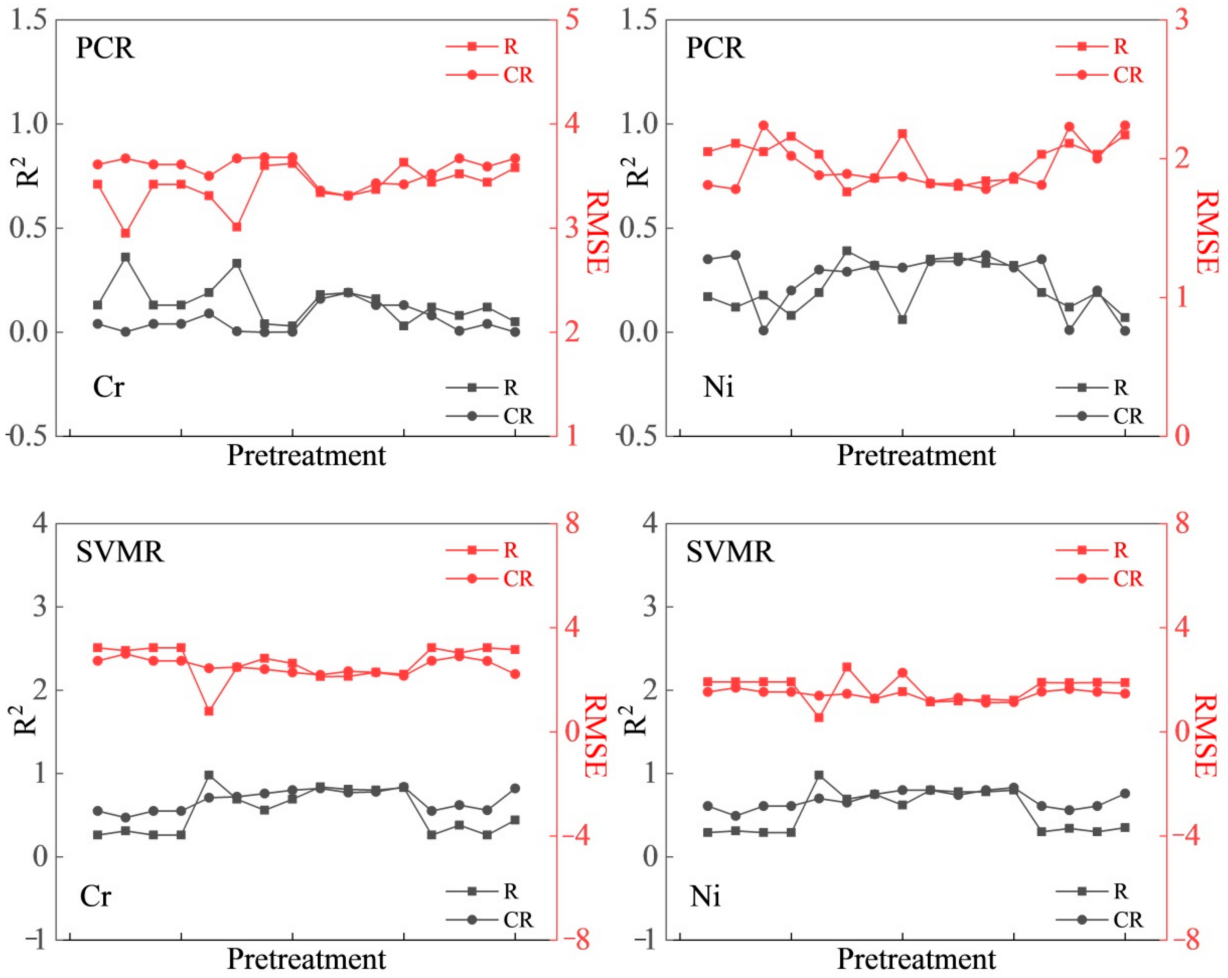
Determination effect of chromium (Cr, mg/kg) and nickel (Ni, mg/kg) elements based on the R and CR of Vis–NIR spectra.

**Figure 4:**
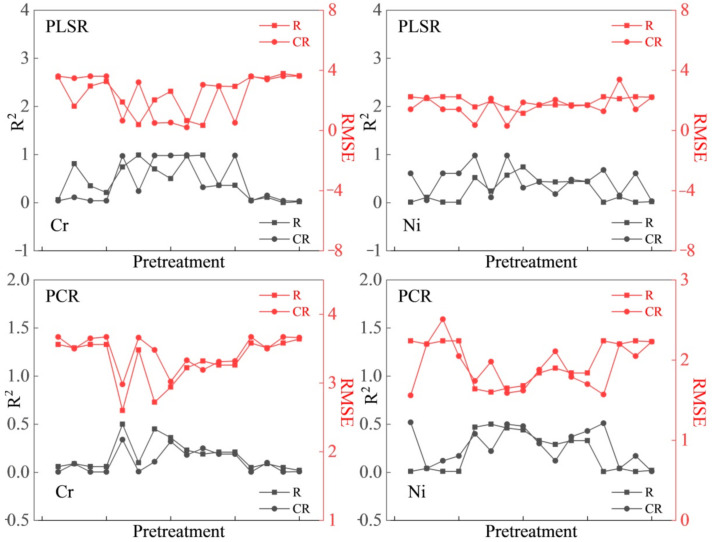

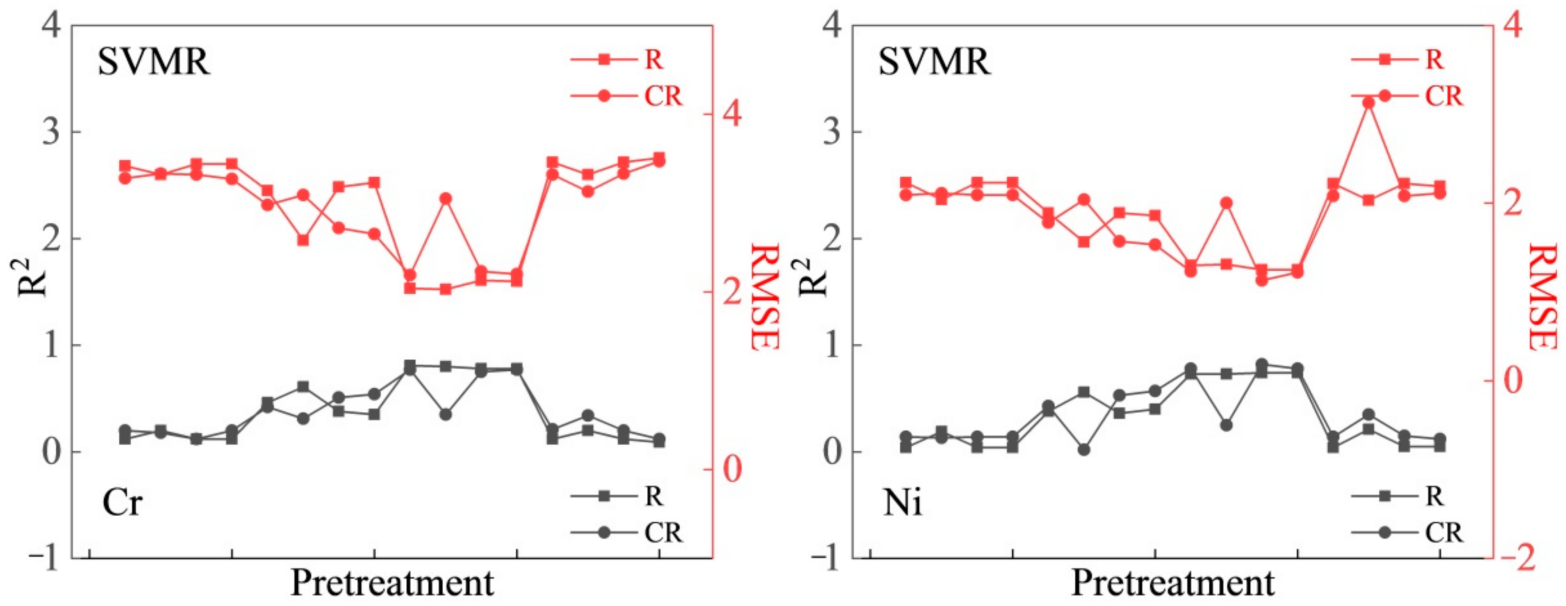
Determination effect of chromium (Cr, mg/kg) and nickel (Ni, mg/kg) elements based on the R and CR of spectral bands associated with SOM.

Taking NOR, MSC, and SNV preprocessing methods and FD, SD, and (log (1/R) spectral transformation data as modeling variables, a heavy metal estimation model was developed using the PLSR, PCR, and SVMR methods. Figure 3 and Figure 4 show plots of R^2^ and RMSE for the determination of the entire data (37 samples) of Cr and Ni concentrations on the basis of R and CR spectra, in which the circle symbol line represents CR, and the square symbol line represents R. CR can effectively enhance the spectral reflectance characteristics of different land types [63]. The stability and accuracy of the model based on CR spectra were found to be significantly higher than that of R. In general, the R^2^ of the two elements in the CR-based model was higher than that of the R-based model, while the RMSE of the CR-based model was lower than that of the R-based model. The results showed that CR can enhance the spectral characteristics and improve the determination accuracy. Therefore, CR data were selected as the basic spectral data in this study. 

#### 3.2.2. Estimation Models Based on Different Preprocessing Methods

Taking NOR, MSC, and SNV preprocessing and FD, SD, and (log (1/R) spectral transformation data of CR spectra as modeling variables, the PLSR, PCR, and SVMR methods were applied to establish a model for determining soil heavy metal concentration. Table 3, Table 4, Table 5 and Table 6 show the determination results of Cr and Ni concentrations with different spectral preprocessing and spectral datasets, respectively. The results of the three spectral transformations showed that the SD transformation is more suitable for the model. Among the three preprocessing methods, the MSC and SNV groups had a significant impact on the determination ability of the model. The MSC and SNV groups exhibited the highest fitting accuracy for Cr and Ni. In addition, the combinations of MSC-SD and SNV-SD showed the highest performance (SOM-based PLSR modeling parameters, MSC-SD (Cr): R^2^ = 0.36/RMSE = 2.95, SNV-SD (Cr): R^2^ = 0.98/RMSE = 0.51; MSC-SD (Ni): R^2^ = 0.48/RMSE = 1.63, SNV-SD (Ni): R^2^ = 0.44/RMSE = 1.68, respectively. SOM-based PCR modeling parameters: MSC-SD (Cr): R^2^ = 0.19/RMSE = 3.31, SNV-SD (Cr): R^2^ = 0.19/RMSE = 3.32; MSC-SD (Ni): R^2^ = 0.37/RMSE = 1.79, SNV-SD (Ni): R^2^ = 0.43/RMSE = 1.70, respectively. SOM based SVMR modeling parameters: MSC-SD (Cr): R^2^ = 0.75/RMSE = 2.23, SNV-SD (Cr): R^2^ = 0.77/RMSE = 2.20; MSC-SD (Ni): R^2^ = 0.82/RMSE = 1.13, SNV-SD (Ni): R^2^ = 0.78/RMSE = 1.22, respectively). In general, in terms of model stability, the R^2^ values of the two elements were higher for the model based on MSC and SNV than that based on NOR, and it was higher for the model based on SD than the model based on FD and log(1/R). In terms of model accuracy, the RMSE values of Cr and Ni elements were lower in the model based on MSC and SNV than in the model based on NOR, and lower in the model based on SD than that based on FD and log(1/R). The optimal model for Cr based on the Vis–NIR dataset and PLSR, PCR, and SVMR is the combination of MSC-SD, SNV-SD, and SNV-SD, respectively. The optimal model for Cr based on the SOM dataset and PLSR, PCR, and SVMR is the combination of SNV-SD, MSC-SD, and SNV-SD, respectively. The optimal model for Ni based on the Vis–NIR dataset and PLSR, PCR, and SVMR is the combination of SNV-SD, MSC-SD, and SNV-SD, respectively. The optimal model for Ni based on the SOM dataset and PLSR, PCR, and SVMR is the combination of MSC-SD, SNV-SD, and MSC-SD, respectively.

#### 3.2.3. Estimation Model Based on Different Modeling Variables

Based on the abovementioned analysis and spectral bands (600–800 nm) associated with SOM and Vis–NIR after CR treatment, the MSC-SD and SNV-SD preprocessing methods were applied to establish models for the determination of soil heavy metal concentrations. Table 7 shows the determination accuracies of the calibration and validation models based on spectral bands associated with SOM and Vis–NIR for Cr and Ni concentrations. 

Regarding model stability, the R^2^ values of the Vis–NIR-based model for Cr and Ni were higher than those of the SOM-based model (Table 7). Regarding model accuracy, the Vis–NIR-based model with PLSR, PCR, and SVMR for Cr showed RMSEC values of 0.46, 3.75, and 3.87 and RMSEV values of 1.56, 2.06 and 4.27, respectively. The SOM model with PLSR, PCR, and SVMR for Cr showed RMSEC values of 0.67, 3.88, and 3.85 and RMSEV values of 1.69, 2.57 and 4.22, respectively. The Vis–NIR-based model with PLSR, PCR, and SVMR for Ni showed RMSEC values of 0.38, 1.76, and 2.27 and RMSEV values of 1.28, 1.99, and 2.52, respectively. The SOM-based model with PLSR, PCR, and SVMR for Ni showed RMSEC values of 0.33, 2.34, and 2.31 and RMSEV values of 1.44, 1.42 and 2.52, respectively. The lower RMSE values of the Vis–NIR-based model indicate its higher accuracy over the SOM-based model. The model for Cr and Ni was sensitive to the Vis–NIR spectral band. The R^2^ value of the PLSR model with Vis–NIR was stable above 0.55 (*p* > 0.05) and the RMSE value was between 0.38 and 1.56. The model had a strong ability to determine the concentrations of the two elements, and the model exhibited greater ability for Cr than Ni. In contrast, the accuracy of determination using the spectral bands associated with SOM was lower. As shown in Table 7, the model accuracies of the different modeling variables were balanced. Models based on the Vis–NIR spectral band were more accurate for Cr and Ni. Stable and highly accurate determination is key to the application of spectroscopy for the determination of soil heavy metal concentration.

#### 3.2.4. Estimation Model Based on Different Statistical Methods 

Based on the abovementioned analysis and the Vis–NIR spectral band after CR treatment, the MSC-SD and SNV-SD preprocessing methods were applied to establish models for the determination of soil heavy metal concentration. Table 7 shows the determination accuracies of the calibration and validation models based on different statistical methods for Cr and Ni concentrations.

Regarding model stability, the R^2^ values of the PLSR-based model for Cr and Ni were higher than those of the PCR- and SVMR-based models, and SVMR showed higher values than PCR. In terms of model accuracy, PLSR, PCR, and SVMR for Cr showed RMSEC values of 0.46, 3.75, and 3.81 and RMSEV values of 1.56, 2.06 and 4.27, respectively. For Ni, PLSR, PCR, and SVMR showed RMSEC values of 0.38, 1.76, and 2.27 and RMSEV values of 1.28, 1.99 and 2.52, respectively. The lower RMSE values of the PLSR-based model indicate its higher accuracy over the PCR- and SVMR-based models. The models for Cr and Ni were sensitive to the PLSR and SVMR statistical methods. The constructed PLSR model was stable with R_c_^2^ and R_V_^2^ values above 0.55 (*p* > 0.05) and highly accurate, with RMSEC and RMSEV values between 0.38 and 1.56. The model had a strong determinative ability for these elements, and the proposed approach can be used to predict the concentrations of these elements with satisfactory precision. The determinative abilities of the three statistical methods follow the order PLSR > SVMR > PCR. In addition, the PCR statistical method showed the lowest accuracy. As shown in Table 7, the model accuracies of the different statistical methods were balanced. The results showed that the models based on PLSR and SVMR were more stable for Cr and Ni concentrations.

Through the statistics obtained from the abovementioned analysis, the Vis–NIR dataset and PLSR model were validated. Furthermore, data from nine sampling points in the study area were used to validate the PLSR estimation model for Cr and Ni concentrations (as shown in Table 8). Regarding model stability, the R^2^ values for Cr and Ni were 0.54 (*p* > 0.05) and 0.57 (*p* > 0.05), respectively. In terms of model accuracy, the RMSEP values for Cr and Ni were 2.02 and 0.02, respectively. The results showed that the PLSR model constructed using Vis–NIR spectra had good quantitative prediction ability.

## 4. Discussion

Preprocessing of soil spectral data is an essential and efficient means for improving the accuracy of hyperspectral modeling [64]. Preprocessing methods exhibit varying performances with different modeling approaches. In this study, taking NOR, MSC, and SNV preprocessing and FD, SD, and (log (1/R) spectral transformation data of CR spectral as modeling variables, a model for determining soil heavy metal concentration was established. Among the three preprocessing methods, the MSC and SNV groups significantly affected the determination ability of the model. Ren et al. constructed the PCR and PLSR prediction model of As and Fe concentrations and OM content using the Vis–NIR spectra of farmland soil in the mining area and soil data as pollution concentration, Fe and OM content, obtained in the laboratory. The research showed that the prediction ability of the model could be significantly improved through MSC, SNV and CR preprocessing [65]. Riedel et al. used 203 soil samples from the German Saxony soil monitoring program covering the period 1998–2013 to test the potential of Vis–NIR and mid-infrared (MIR) in the quantitative prediction of soil properties. They that showed spectroscopy can provide reliable information of soil metal content in a rapid manner, and two preprocessing methods, MSC and SNV transformation, can improve the performance of the model [66]. Zheng et al. used the PLSR method to establish the relationship between reflectance spectral and As content in soil. Compared with other methods, they showed that MSC provides a more accurate prediction (R^2^ = 0.711, RMSE = 1.613) [67]. Wu et al. found that baseline smoothing and MSC pretreatment of MID spectral data significantly improve the prediction ability of the model for heavy metal content in off-site soil samples [68] by eliminating the influence of light scattering and sample thickness. The results of this study are very close to those of Ren, Riedel, Zheng, and Wu [64,65,66,67]. The prediction ability of different soil elements based on different preprocessing at different study areas was investigated. MSC and SNV transformation were found to improve the performance of the model. Light scattering effects and baseline shifts of the spectra are among the main factors affecting the spectroradiometer signal in the Vis–NIR [69]. By effectively reducing systematic errors and background noise of the whole sample, the MSC and SNV methods improve the SNR [70].

The limitations of statistical models vary among different soil types, different methods of data preprocessing, different spectral resolutions, different band ranges used, or different forms of transformations, leading to large differences in the accuracy of the same model or different best models for determination. In general, the PLSR algorithm is superior to PCR and SVMR and can monitor the concentration of heavy metals in soil with good results. Compared with the SVMR and PCR algorithms, PLSR firstly extracts principal component information of both spectral band and heavy metal concentration variable matrices and uses a constraint equation in the process of dimensionality reduction to ensure the maximum correlation between spectral band and heavy metal concentration variable component information. Although PCR also involves the extraction of principal components to reduce dimensionality, it only extracts the information of the spectral band variable matrix, without considering the information of the heavy metal concentration variable matrix and does not reduce the dimensionality of the heavy metal concentration variable matrix. Therefore, further optimization operations are required. Some scholars [71] also found that the PLSR method provides better results than the PCR method because the latent variable of PLSR contains information about the OM content. The SVMR method is a nonlinear modeling method, while the PLSR and PCR methods are linear methods. In this study, radial basis functions were mainly used for nonlinear modeling, but the results were not satisfactory in combination with the experimental data, mainly because the RMSE values were large. Choe et al. [72] monitored heavy metal pollution in river sediments in Rodalquilar, southeastern Spain; using a combination of geochemistry, ground spectral parameters, and hyperspectral remote sensing, they obtained parameters from spectral changes related to heavy metals in soil. Ground spectral parameters obtained from the spectral absorption characteristics were found to have potential applicability in analyzing the spatial distribution of heavy metal elements, while the spectral characteristics of soil were not obvious. In terms of scores, PLSR modeling is highly advantageous for making predictions. Kooristra et al. successfully predicted the composition and heavy metal content of beach soil using a PLSR model established using soil Vis–NIR, and pointed out that PLSR method is an effective approach toward predicting the heavy metal content of soil using spectral methods [8].

Compared with the SVMR and PCR methods, the PLSR method uses fewer latent variables, but the model has higher fitting and stability, and has stronger determinative ability, indicating that the latent variables used by the PLSR method contain more soil physicochemical information. Wang [73] used the PLSR method to compare and analyze various spectral indices, and showed that the reciprocal logarithm spectra had the best determinative ability, especially with the detection accuracy of Cd and Pb exceeding 0.82. McDowell et al. also found that spectral characteristic variables related to various organic components and silicate minerals were fully utilized in the PLSR modeling and determination process [74]. Malley [75] pointed out a linear relationship between the absorbance of the NIR spectrum and the concentration of substances. However, some scholars have reported different findings. Shao et al. found that the determination result of the least squares support vector machine (LS-SVM) is better than that of PLSR when using NIR spectra to determine soil NPK [76]. It is speculated that LS-SVM uses the nonlinear information of spectral data to improve the determination accuracy. Evaluating different spectral datasets and different statistical methods, PLSR modeling was found to be very beneficial to the prediction of soil composition and heavy metal concentration. No modeling method is universal, and a model that performs well in one application may not be suitable for another. Therefore, when using spectral data to determine soil properties, the optimal modeling regression method varies across study areas, spectral ranges, and target components.

Soil heavy metals and components, such as SOM, clay minerals, and iron and manganese oxides, exhibit obvious spectral characteristics [23,24]. There is a significant correlation between heavy metals and soil spectral characteristics, such as OM, clay, and Fe [8,20]. Therefore, these properties may play a bridging role in the determination of soil heavy metal concentrations using Vis–NIR reflectance. By selecting characteristic bands, the original spectral information can be well retained and the relationship between soil spectral characteristics and SOM and heavy metals can be reflected more accurately. According to the crystal field theory [77], transition elements with unfilled d-shells, such as Ni, Cu, and Cr, can exhibit absorption characteristics in the Vis–NIR spectral regions. Iron oxides, clay minerals, water content, and SOM are active in Vis–NIR spectral regions [21,22]. The results in Table 7 show that the models for Cr and Ni are sensitive to the Vis–NIR spectral band. The model based on Vis–NIR exhibited stable R^2^ values above 0.98 and RMSE values ranging from 0.07 to 0.34, suggesting a strong determinative ability for Cr and Ni. These results confirm that the Vis–NIR technique can improve the accuracy of Cr and Ni estimation models, and that the Vis–NIR technique has strong potential for the simultaneous monitoring and estimation of different species of heavy metals in soils, providing an effective method for large-scale and long-term monitoring of soil heavy metal contamination. Future studies could consider other factors such as Fe–Mn oxide and extract multi-factor characteristic bands to construct multi-spectral transformation indices and estimation models. In the future, the SNV–SD–PLSR method can be verified and promoted through application to other study areas, such as field spectral analysis, and even to UAV and satellite remote sensing data.

## 5. Conclusions

This study evaluated three preprocessing methods (NOR, MSC, and SNV), three spectral transformations (FD, SD, and LOG), and three statistical methods (PLSR, PCR, and SVMR). This approach can enhance variable information, reduce model errors, and improve the accuracy and stability of the model. The mechanism of determining heavy metal concentration was systematically analyzed, the relationship between heavy metal concentration and spectral analysis in the soil around a mining area was determined, and different preprocessing and statistical methods were compared to provide important scientific support for heavy metal pollution research. It is considered that the absorption spectral band at 600–800 nm was associated with SOM. The CR data were selected as the basic spectral data, and MSC–SD and SNV–SD were found to be the best among the 16 preprocessing methods for determining Cr and Ni concentrations. The estimation models for Cr and Ni were sensitive to the Vis–NIR spectral band. The R^2^ value of the PLSR model built using Vis–NIR was stable above 0.55, the RMSE value was between 0.38 and 1.56, and the model had a strong ability to determine the concentration of two elements, in the order of Cr > Ni. In contrast, the accuracy of determination using the spectral bands associated with SOM is lower. The performances of the three statistical methods are as follows: PLSR > SVMR > PCR, and the accuracy of determination using the PCR statistical method is lower. The estimation models based on the PLSR and SVMR statistical methods are more stable for Cr and Ni concentrations. In the future, the SNV–SD–PLSR method could be applied to other study areas, from field spectral to even UAV and satellite remote sensing data for verification and promotion.

## Figures and Tables

**Table 1:** Combination method of spectral data preprocessing and spectral transformation.

Preprocessing and Spectral Transformation			
SG + R	SG + R + FD	SG + R + SD	SG + R + LOG
SG + NOR	SG + NOR + FD	SG + NOR + SD	SG + NOR + LOG
SG + MSC	SG + MSC + FD	SG + MSC + SD	SG + MSC + LOG
SG + SNV	SG + SNV + FD	SG + SNV + SD	SG + SNV + LOG

SG: Savitzky–Golay, R: reflectance, NOR: normalization, MSC: multiplicative scatter correction, SNV: standard normal variate, FD: first derivative, SD: second derivative, LOG: reciprocal logarithm.

**Table 2:** Statistical results of heavy metal elements, SOM, and water content for soil samples.

Elements	Calibration/Validation Set		Validation Statistics		Soil Organic Matter (%)	Water Content (g)
	Cr (mg/kg)	Ni (mg/kg)	Cr (mg/kg)	Ni (mg/kg)		
Mean	16.59	5.78	22.02	7.49	2.93	5.06
Std.	3.73	2.28	4.70	2.07	1.52	3.8
Kurtosis	0.11	0.7	−0.58	−0.09	−0.18	−0.01
Skewness	−0.24	−0.56	−0.54	−0.37	0.2	0.52
Min.	8.02	0.01	13.52	3.88	0.04	0
Max.	24.12	10.22	27.25	10.69	6.82	15.5
*n*	37	37	9	9	37	37
CV	0.22	0.39	0.21	0.28	0.52	0.75
K-S test Asymp.Sig.	0.2	0.2	0.2	0.2	0.2	0.2
Background value	21.15	10.07	21.15	10.07		
Secondary standard (pH > 7.5)	250	60	250	60		

*n*: number, CV: coefficient of variation.

**Table 3:** Determination accuracies of Cr concentrations based on Vis–NIR spectral bands.

Preprocessing	PLSR		PCR		SVMR	
	RMSE	R^2^	RMSE	R^2^	RMSE	R^2^
SG + CR	3.3	0.19	3.61	0.04	2.73	0.55
SG + CR + NOR	3.3	0.2	3.67	0.002	3	0.47
SG + CR + MSC	3.3	0.2	3.61	0.04	2.73	0.55
SG + CR + SNV	3.3	0.19	3.61	0.04	2.73	0.55
SG + CR + FD	1.25	0.88	3.5	0.09	2.44	0.71
SG + CR + NOR + FD	3.03	0.32	3.67	0.004	2.49	0.72
SG + CR + MSC + FD	1.17	0.9	3.68	0.0002	2.41	0.76
SG + CR + SNV + FD	0.25	0.99	3.68	0.002	2.28	0.8
SG + CR + SD	0.45	0.98	3.36	0.16	2.19	0.82
SG + CR + NOR + SD	1.64	0.8	3.31	0.19	2.33	0.77
SG + CR + MSC + SD	0.34	0.99	3.43	0.13	2.28	0.78
SG + CR + SNV + SD	2.76	0.44	3.42	0.13	2.15	0.84
SG + CR + LOG	3.3	0.19	3.52	0.08	2.73	0.55
SG + CR + NOR + LOG	1.09	0.91	3.67	0.006	2.9	0.62
SG + CR + MSC + LOG	3.3	0.19	3.59	0.04	2.72	0.56
SG + CR + SNV + LOG	1.43	0.85	3.67	0.001	2.22	0.82

PLSR: partial least squares regression, PCR: principal component regression, SVMR: support vector machine regression, RMSE: root-mean-square error, R^2^: coefficient of determination, SG: Savitzky–Golay, CR: continuum-removal, NOR: normalization, MSC: multiplicative scatter correction, SNV: standard normal variate, FD: first derivative, SD: second derivative, LOG: reciprocal logarithm.

**Table 4:** Determination accuracies of Ni concentrations based on Vis–NIR spectral bands.

Preprocessing	PLSR		PCR		SVMR	
	RMSE	R^2^	RMSE	R^2^	RMSE	R^2^
SG + CR	1.82	0.34	1.81	0.35	1.53	0.61
SG + CR + NOR	1.71	0.42	1.78	0.37	1.69	0.49
SG + CR + MSC	1.82	0.34	2.24	0.008	1.53	0.61
SG + CR + SNV	1.82	0.34	2.02	0.2	1.53	0.61
SG + CR + FD	0.27	0.99	1.88	0.3	1.39	0.7
SG + CR + NOR + FD	0.21	0.99	1.89	0.29	1.46	0.65
SG + CR + MSC + FD	1.29	0.67	1.86	0.32	1.28	0.75
SG + CR + SNV + FD	0.4	0.97	1.87	0.31	2.27	0.8
SG + CR + SD	0.25	0.99	1.82	0.34	1.17	0.8
SG + CR + NOR + SD	1.59	0.5	1.82	0.34	1.31	0.74
SG + CR + MSC + SD	0.19	0.99	1.78	0.37	1.12	0.8
SG + CR + SNV + SD	0.07	0.99	1.87	0.31	1.14	0.83
SG + CR + LOG	1.82	0.34	1.81	0.35	1.54	0.61
SG + CR + NOR + LOG	1.98	0.22	2.23	0.01	1.64	0.56
SG + CR + MSC + LOG	1.82	0.34	2	0.2	1.53	0.61
SG + CR + SNV + LOG	1.48	0.57	2.24	0.006	1.47	0.76

**Table 5:** Determination accuracies of Cr concentrations based on spectral bands associated with SOM.

Preprocessing	PLSR		PCR		SVMR	
	RMSE	R^2^	RMSE	R^2^	RMSE	R^2^
SG + CR	3.61	0.04	3.67	0.004	3.28	0.2
SG + CR + NOR	3.48	0.11	3.5	0.09	3.33	0.18
SG + CR + MSC	3.61	0.04	3.65	0.004	3.32	0.12
SG + CR + SNV	3.61	0.04	3.67	0.004	3.27	0.2
SG + CR + FD	0.65	0.97	2.98	0.34	2.98	0.42
SG + CR + NOR + FD	3.21	0.24	3.66	0.008	3.09	0.31
SG + CR + MSC + FD	0.5	0.98	3.48	0.11	2.72	0.51
SG + CR + SNV + FD	0.53	0.98	3.02	0.32	2.65	0.54
SG + CR + SD	0.21	0.99	3.33	0.18	2.19	0.77
SG + CR + NOR + SD	3.04	0.32	3.19	0.25	3.05	0.35
SG + CR + MSC + SD	2.95	0.36	3.31	0.19	2.23	0.75
SG + CR + SNV + SD	0.51	0.98	3.32	0.19	2.2	0.77
SG + CR + LOG	3.61	0.04	3.67	0.004	3.32	0.21
SG + CR + NOR + LOG	3.39	0.15	3.5	0.1	3.13	0.34
SG + CR + MSC + LOG	3.61	0.04	3.67	0.004	3.33	0.2
SG + CR + SNV + LOG	3.62	0.03	3.66	0.007	3.47	0.12

**Table 6:** Determination accuracies of Ni concentrations based on spectral bands associated with SOM.

Preprocessing	PLSR		PCR		SVMR	
	RMSE	R^2^	RMSE	R^2^	RMSE	R^2^
SG + CR	1.41	0.61	1.56	0.52	2.09	0.14
SG + CR + NOR	2.19	0.05	2.2	0.04	2.11	0.13
SG + CR + MSC	1.41	0.61	2.51	0.12	2.09	0.14
SG + CR + SNV	1.41	0.61	2.05	0.17	2.09	0.14
SG + CR + FD	0.36	0.98	1.74	0.4	1.78	0.43
SG + CR + NOR + FD	2.12	0.11	1.98	0.22	2.04	0.02
SG + CR + MSC + FD	0.3	0.98	1.59	0.5	1.57	0.53
SG + CR + SNV + FD	1.87	0.31	1.62	0.48	1.53	0.57
SG + CR + SD	1.7	0.43	1.88	0.3	1.23	0.78
SG + CR + NOR + SD	2.04	0.18	2.11	0.12	2	0.25
SG + CR + MSC + SD	1.63	0.48	1.79	0.37	1.13	0.82
SG + CR + SNV + SD	1.68	0.44	1.7	0.43	1.22	0.78
SG + CR + LOG	1.28	0.68	1.57	0.51	2.08	0.14
SG + CR + NOR + LOG	3.39	0.15	2.2	0.04	3.13	0.35
SG + CR + MSC + LOG	1.4	0.61	2.05	0.17	2.08	0.15
SG + CR + SNV + LOG	2.21	0.03	2.23	0.01	2.11	0.12

**Table 7:** Determination accuracies of Cr, and Ni concentrations based on spectral bands associated with SOM and Vis–NIR.

Dataset	Statistical Method	Elements	Calibration (*n* = 27)		Validation (*n* = 10)	
			RMSEC	R_C_^2^	RMSEV	R_V_^2^
Vis–NIR	PLSR	Cr	0.46	0.99	1.56	0.66
		Ni	0.38	0.97	1.28	0.55
	PCR	Cr	3.75	0.12	2.06	0.42
		Ni	1.76	0.35	1.99	0.33
	SVMR	Cr	3.81	0.68	4.27	0.38
		Ni	2.27	0.61	2.52	0.17
SOM	PLSR	Cr	0.67	0.97	1.69	0.61
		Ni	0.33	0.98	1.44	0.43
	PCR	Cr	3.88	0.06	2.57	0.09
		Ni	2.34	0.05	1.42	0.45
	SVMR	Cr	3.85	0.53	4.22	0.36
		Ni	2.31	0.59	2.52	0.25

**Table 8:** Validation of the models for prediction of soil Cr, and Ni concentrations based on Vis–NIR.

Statistical Method	Elements	Validation (*n* = 9)	
		RMSEP	R_P_^2^
PLSR	Cr	2.02	0.54
	Ni	0.02	0.57

RMSEP: root-mean-square error of prediction.

## Data Availability

Not applicable.

## References

[1] Wu J., Long J., Liu L., Li J., Liao H., Zhang M., Zhao C., Wu Q. (2018). Risk Assessment and Source Identification of Toxic Metals in the Agricultural Soil around a Pb/Zn Mining and Smelting Area in Southwest China. Int. J. Environ. Res. Public Health.

[2] Jamal A., Delavar M.A., Naderi A., Nourieh N., Medi B., Mahvi A.H. (2019). Distribution and health risk assessment of heavy metals in soil surrounding a lead and zinc smelting plant in Zanjan, Iran. Hum. Ecol. Risk. Assess..

[3] Kasemodel M.C., Sakamoto I.K., Varesche M.B.A., Rodrigues V.G.S. (2019). Potentially toxic metal contamination and microbial community analysis in an abandoned pb and zn mining waste deposit. Sci. Total Environ..

[4] Karbassi S., Nasrabadi T., Shahriari T. (2016). Metallic pollution of soil in the vicinity of National Iranian Lead and Zinc (NILZ) Company. Environ. Earth Sci..

[5] Huang B., Guo Z.H., Tu W.J., Peng C., Xiao X., Zeng P., Liu Y., Wang M., Xiong J. (2018). Geochemistry and ecological risk of metal(loid) s in overbank sediments near an abandoned lead/zinc mine in Central South China. Environ. Earth Sci..

[6] Wang Y., Yang L., Kong L., Liu E., Wang L., Zhu J. (2015). Spatial distribution, ecological risk assessment and source identification for heavy metals in surface sediments from dongping lake, shandong, east china. Catena.

[7] Gan F., Fang W., Wang X., Yang S., Zheng H. (2008). The heavy metal contamination in soil-potato and pea of tin tailings. Ecol. Env..

[8] Kooistra L., Wehrens R., Leuven R.S.E.W., Buydens L.M.C. (2001). Possibilities of visible-near-infrared spectroscopy for the assessment of soil contamination in river floodplains. Anal. Chim. Acta.

[9] Rossel R.A.V., Walvoort D.J.J., Mcbratney A.B., Janik L.J., Skjemstad J.O. (2006). Visible, near infrared, mid infrared or combined diffuse reflectance spectroscopy for simultaneous assessment of various soil properties. Geoderma.

[10] Liu F., Zhang F., Jin Z., He Y., Fang H., Ye Q., Zhou W. (2008). Determination of acetolactate synthase activity and protein content of oilseed rape (*Brassica napus* L.) leaves using visible/near-infrared spectroscopy. Anal. Chim. Acta.

[11] Malley D.F., Williams P.C. (1997). Use of near-infrared reflectance spectroscopy in prediction of heavy metals in freshwater sediment by their association with organic matter. Environ. Sci. Technol..

[12] Zhu Z., Shen H., Wang N., Zhu R. (2018). Transient measure technique for excitation temperature and radiation temperature based on multi-spectral method. Spectrosc. Spectr. Anal..

[13] Tian Q., Min X. (1998). Advances in study on vegetation indices. Adv. Earth. Sci..

[14] Zhang T., Zhao Y., An H., Chen X. (2011). Selection of ETM+ Remote Sensing Image Optimum Waveband Combination in Information Extraction of Sinking Sandy Land-The Case in Xiwu Flag, Xilin Gol League, Inner Mongolia. Sci. Technol. Rev..

[15] Nicola B.M., Beullens K., Bobelyn E., Peirs A., Saeys W., Theron K.I., Lammerty J. (2007). Nondestructive measurement of fruit and vegetable quality by means of nir spectroscopy: A review. Postharvest Biol. Tech..

[16] Shamsoddini A., Raval S., Taplin R. (2014). Spectroscopic analysis of soil metal contamination around a derelict mine site in the blue mountains, Australia. ISPRS J. Photogramm..

[17] Wang L., Bai J., Lei Y., Wang H. (2011). Effect on retrieval precision for corn N content by spectrum data transformation. Remote Sens. Technol. Appl..

[18] Mashimbye Z.E. (2012). Model-based integrated methods for quantitative estimation of soil salinity from hyperspectral remote sensing data: A case study of selected South African. Pedosphere.

[19] Lu Q., Wang S., Bai X., Liu F., Wang M., Wang J., Tian S. (2019). Rapid inversion of heavy metal concentration in karst grain producing areas based on hyperspectral bands associated with soil components. Microchem. J..

[20] Liu Y. (2020). Inversion of Heavy Metals in Farmland Surface Soil Based on Vis-NIR Spectrum.

[21] Wu Y., Chen J., Wu X., Tian Q., Ji J., Qin Z. (2005). Possibilities of reflectance spectroscopy for the assessment of contaminant elements in suburban soils. Appl. Geochem..

[22] Rathod P.H., Rossiter D., Noomen M., van der Meer F.D. (2013). Proximal spectral sensing to monitor phytoremediation of metal-contaminated soils. Int. J. Phytorem..

[23] Viscarra Rossel R.A., Behrens T. (2010). Using data mining to model and interpret soil diffuse reflectance spectra. Geoderma.

[24] Bradl H.B. (2004). Adsorption of heavy metal ions on soils and soils constituents. J. Colloid Interface Sci..

[25] Kemper T., Sommer S. (2002). Estimate of heavy metal contamination in soils after a mining accident using reflectance spectroscopy. Environ. Sci. Technol..

[26] Sorenson P.T., Quideau S.A., Rivard B. (2018). High resolution measurement of soil organic carbon and total nitrogen with laboratory imaging spectroscopy. Geoderma.

[27] Sun W., Zhang X., Sun X., Sun Y., Cen Y. (2018). Predicting nickel concentration in soil using reflectance spectroscopy associated with organic matter and clay minerals. Geoderma.

[28] Sun W., Zhang X. (2017). Estimating soil zinc concentrations using reflectance spectroscopy. Int. J. Appl. Earth Obs. Geoinf..

[29] Moron A., Cozzolino D. (2003). Exploring the use of near infrared reflectance spectroscopy to study physical properties and microelements in soils. J. Near Infrared Spec..

[30] Grzegorz S., Mccarty G.W., Stuczynski T.I., Reeves J.B. (2004). Near- and mid-infrared diffuse reflectance spectroscopy for measuring soil metal content. J. Environ. Qual..

[31] Zhang X., Sun W., Cen Y., Zhang L., Wang N. (2018). Predicting cadmium concentration in soils using laboratory and field reflectance spectroscopy. Sci. Total Environ..

[32] Covelo E.F., Vega F.A., Andrade M.L. (2007). Simultaneous sorption and desorption of Cd, Cr, Cu, Ni, Pb, and Zn in acid soils II. Soil ranking and influence of soil characteristics. J. Hazard. Mater..

[33] Covelo E.F., Vega F.A., Andrade M.L. (2007). Competitive sorption and desorption of heavy metals by individual soil components. J. Hazard. Mater..

[34] Alloway B.J. (1995). Heavy Metals in Soils. Heavy Metals in Soils.

[35] BenDor E., Inbar Y., Chen Y. (1997). The reflectance spectra of organic matter in the visible near-infrared and short wave infrared region (400–2500 nm) during a controlled decomposition process. Remote Sens. Environ..

[36] Wu Y., Chen J., Ji J., Gong P., Liao Q., Tian Q., Ma H. (2007). A mechanism study of reflectance spectroscopy for investigating heavy metals in soils. Soil Sci. Soc. Am. J..

[37] Shibusawa S., Anom S.W.I., Sato S., Sasao A., Hirako S., Grenier G., Blackmore S. (2001). Soil mapping using the real-time soil spectrophotometer. Proceedings of the Third European Conference on Precision Agriculture.

[38] Chang C.W., Laird D.A., Mausbach M.J., Hurburgh C.R. (2001). Near-infrared reflectance spectroscopy–principal component regression analyses of soil properties. Soil Sci. Soc. Am. J..

[39] Lucà F., Conforti M., Matteucci G., Buttafuoco G. Prediction of organic carbon and nitrogen in forest soil using laboratory visible and near infrared spectroscopy. Proceedings of the 1st Conference on Proximal Sensing Supporting Precision Agriculture-Held at Near Surface Geoscience.

[40] Fidêncio P.H., Poppi R.J., De Andrade J.C. (2002). Determination of organic matter in soils using radial basis function networks and near infrared spectroscopy. Anal. Chim. Anal. Chim. Acta.

[41] Ramirez-Lopez L., Schmidt K., Behrens T., Van Wesemael B., Demattê J.A.M., Scholten T. (2014). Sampling optimal calibration sets in soil infrared spectroscopy. Geoderma.

[42] Vasques G.M., Grunwald S., Sickman J.O. (2008). Comparison of multivariate methods for inferential modeling of soil carbon using visible/near-infrared spectra. Geoderma.

[43] Vapnik V.N. (1995). The Nature of Statistical Learning Theory. Information Science and Statistics.

[44] Computer Network Information Center, Chinese Academy of Science, Geospatial Data Cloud. http://www.gscloud.cn/sources/accessdata/411?pid=263.

[45] Li F., Cai Y., Zhang J. (2018). Spatial characteristics, health risk assessment and sustainable management of heavy metals and metalloids in soils from Central China. Sustainability.

[46] Jiang Y., Ruan X., Yang L., He W., Jiao Y., Wang H. (2017). Distribution of Hg, As and Sb concentrations in urban soil profiles of Kaifeng City, Henan Province. Environ. Chem..

[47] Bernalte E., Marin Sanchez C., Pinilla Gil E. (2013). High-Throughput Mercury Monitoring in Indoor Dust Microsamples by Bath Ultrasonic Extraction and Anodic Stripping Voltammetry on Gold Nanoparticles-Modified Screen-Printed Electrodes. Electroanalysis.

[48] Pueyo M., Mateu J., Rigol A., Vidal M., López-Sánchez J.F., Rauret G. (2008). Use of the modified BCR three-step sequential extraction procedure for the study of trace element dynamics in contaminated soils. Environ. Pollut..

[49] Chen T., Chang Q., Clevers J.G., Kooistra L. (2015). Rapid identification of soil cadmium pollution risk at regional scale based on visible and near-infrared spectroscopy. Environ. Pollut..

[50] Kokaly R.F., Clark R.N. (1999). Spectroscopic determination of leaf biochemistry using band-depth analysis of absorption features and stepwise multiple linear regression. Remote Sens. Environ..

[51] Savitzky A., Golay M.J.E. (1964). Smoothing and differentiation of data by simplified least squares procedures. Anal. Chem..

[52] Barnes R.J., Dhanoa M.S., Lister S.J. (1989). Standard normal variate transformation and detrending of near infrared diffuse reflectance spectra. Appl. Spectrosc..

[53] Chen J., Iyo C., Teradab F.J. (2002). Effect of multiplicative scatter correction on wavelength selection for near infrared calibration to determine fat content in raw milk. Near Infrared Spectrosc..

[54] Udelhoven T., Emmerling C., Jarmer T. (2003). Quantitative analysis of soil chemical properties with diffuse reflectance spectrometry and partial least-square regression: A feasibility study. Plant Soil.

[55] Christy C.D. (2008). Real-time measurement of soil attributes using on-the-go near infrared reflectance spectroscopy. Comput. Electron. Agric..

[56] Guo Y., Ji W., Wu H., Shi Z. (2013). Estimation and mapping of soil organic matter based on Vis-NIR reflectance spectroscopy. Spectrosc. Spect. Anal..

[57] Xu B., Ji G., Zhu Y. (1991). A preliminary research of geographic regionalization of China land background and spectral reflectance characteristics of soils. J. Remote Sens.

[58] Wang K., Guo D., Zhang Y., Deng L., Xie R., Lv Q., Yi S., Zheng Y., Ma Y., He S. (2019). Detection of Huang long bing (citrus greening) based on hyperspectral image analysis and PCR. Front. Agric. Sci. Eng..

[59] Su H., Chong C., Yang L. (2014). Research on The Method of Water Depth Inversion of Hyperspectral Image Based on SVR. J. New IND..

[60] Tang Q., Feng M. (2007). DPS Data Processing System.

[61] Liu F., He Y., Wang L. (2008). Comparison of calibrations for the determination of soluble solids content and pH of rice vinegars using visible and short-wave near infrared spectroscopy. Anal. Chim. Acta.

[62] Gu Y., Zhang T., Bai H. (1995). Qualitative Classification of soil background value in Inner Mongolia. Inner Mongolia Environ. Prot..

[63] Wei L., Pu H., Wang Z., Yuan Z., Yan X., Cao L. (2020). Estimation of Soil Arsenic Content with Hyperspectral Remote Sensing. Sensors.

[64] Ladoni M., Bahrami H.A., Alavipanah S.K., Norouzi A.A. (2010). Estimating soil organic carbon from soil reflectance: A review. Precis. Agric..

[65] Ren H., Zhuang D., Qiu D., Pan J. (2009). Analysis of Visible and Near-Infrared Spectra of as—Contaminated Soil in Croplands Beside Mines. Spectrosc. Spect. Anal..

[66] Riedel F., Denk M., Müller I., Barth N., Gläßer C. (2017). Prediction of soil parameters using the spectral range between 350 and 15,000nm: A case study based on the Permanent Soil Monitoring Program in Saxony, Germany. Geoderma.

[67] Zheng G.H., Zhou S.L., Wu S.H. (2011). Prediction of as in soil with reflectance spectroscopy. Spectrosc. Spect. Anal..

[68] Wu D.W., Wu Y.Z., Ma H.R. (2010). Study on the prediction of soil heavy metal elements content based on mid-infrared diffuse reflectance spectra. Spectrosc. Spect. Anal..

[69] Rinnan S., Berg F.V.D., Engelsen S.B. (2009). Review of the most common pre-processing techniques for near-infrared spectra. TRAC-TREND. Anal. Chem..

[70] Mccarty G.W., Reeves J.B., Reeves V.B., Follett R.F., Kimble J.M. (2002). Mid-infrared and near-infrared diffuse reflectance spectroscopy for soil carbon measurement. Soil Sci. Soc. Am. J..

[71] Zeng Y., Lu Y., Du C., Zhou J. (2014). Applying infrared photoacoustic spectroscopy and support vector machine model to quantify soil organic matter content. Acta Pedolog. Sinica.

[72] Choe E., Meer F.V.D., Ruitenbeek F.V., Werff H.V.D., Smeth B.D., Kim K.W. (2008). Mapping of heavy metal pollution in stream sediments using combined geochemistry, field spectroscopy, and hyperspectral remote sensing: A case study of the rodalquilar mining area, se spain. Remote Sens. Environ..

[73] Wang L., Lin Q., Jia D., Shi H., Huang X. (2007). Study on the Prediction of Soil Heavy Metal Elements Content Based on Reflectance Spectra. J. Remote Sens..

[74] McDowell M.L., Bruland G.L., Deenik J.L., Grunwald S., Knox N.M. (2012). Soil total carbon analysis in Hawaiian soils with visible, near-infrared and mid-infrared diffuse reflectance spectroscopy. Geoderma.

[75] Malley D.F., Lockhart L., Wilkinson P., Hauser B. (2000). Determination of carbon, carbonate, nitrogen, and phosphorus in freshwater sediments by near-infrared reflectance spectroscopy: Rapid analysis and a check on conventional analytical methods. J. Paleolimnal..

[76] Shao Y., He Y. (2011). Nitrogen, phosphorus, and potassium prediction in soils, using infrared spectroscopy. Soil Res..

[77] Burns R.G. (1993). Mineralogical Applications of Crystal Field Theory 5.

